# Baicalin enhances antioxidant, inflammatory defense, and microbial diversity of yellow catfish (*Pelteobagrus fulvidraco*) infected with *Aeromonas hydrophila*

**DOI:** 10.3389/fmicb.2024.1465346

**Published:** 2024-09-20

**Authors:** Pupu Yan, Jiali Liu, Yongxi Huang, Tilin Yi, Heng Zhang, Gang Dai, Xiong Wang, Zhenzhen Gao, Bin He, Weili Guo, Yingbing Su, Liwei Guo

**Affiliations:** ^1^Engineering Research Center of Ecology and Agricultural Use of Wetland, Ministry of Education, Yangtze University, Jingzhou, Hubei, China; ^2^College of Animal Science and Technology, Yangtze University, Jingzhou, Hubei, China; ^3^Jingzhou Taihugang Aquatic Technology Co., LTD, Hubei, China; ^4^Jingzhou Mingde Technology Co., LTD, Hubei, China; ^5^College of Animal Husbandry and Veterinary Medicine, Jiangsu Vocational College of Agriculture and Forestry, Jurong, Jiangsu, China; ^6^Wuhan city Academy of Agricultural Sciences Institute of Animal Husbandry and Veterinary, Wuhan, China; ^7^NO. 6 Mildle School of Shahe, Xingtai, Hebei, China

**Keywords:** Baicalin, *Aeromonas hydrophila*, antioxidation, immunoenhancement, gut microbiota

## Abstract

**Introduction:**

The aim of this research was to clarify the mechanism through which baicalin exerts its inhibitory effects on *Aeromonas hydrophila* infection.

**Methods:**

The antibacterial efficacy of baicalin was assessed by determining its minimum inhibitory concentration (MIC) against *A. hydrophila*. Various parameters, including the growth curve, cell wall integrity, biofilm formation, AKP content, and morphological alterations of *A. hydrophila*, were analyzed. In vivo experiments involved the administration of *A. hydrophila* 4 h postintraperitoneal injection of varying doses of baicalin to induce infection, with subsequent monitoring of mortality rates. After a 3 d period, liver, spleen, and intestinal tissues were harvested to evaluate organ indices, antioxidant and immune parameters, as well as intestinal microbial composition.

**Results:**

The findings indicated that baicalin treatment resulted in the disruption of the cell wall of *A. hydrophila*, leading to the loss of its normal structural integrity. Furthermore, baicalin significantly inhibited biofilm formation and facilitated the release of intracellular proteins (*P* < 0.05). In vivo, baicalin enhanced the survival rates of yellow catfish infected with *A. hydrophila*. Compared to the control group, the liver index of yellow catfish was elevated, while the spleen and intestinal indices were reduced in the baicalin-treated group (*P* < 0.05). Additionally, baicalin at an appropriate dosage was found to increase levels of SOD, GSH, CAT, ACP, and AKP in yellow catfish (*P* < 0.05), while simultaneously decreasing MDA accumulation and the mRNA expression of inflammatory markers such as Keap1, IL1, IFN-γ, and TNF-α, (*P* < 0.05). Moreover, baicalin significantly enhanced the operational taxonomic unit (OTU) count in *A. hydrophila*-infected yellow catfish (*P* < 0.05), restoring the abundance of Barnesiellaceae, Enterobacteriaceae, Plesiomonas, and *UBA1819* (*P* < 0.05).

**Discussion:**

In summary, baicalin demonstrates the potential to improve the survival rate of yellow catfish subjected to *A. hydrophila* infection, augment antioxidant and immune responses, mitigate inflammation, and enhance intestinal microbial diversity.

## 1 Introduction

*Aeromonas hydrophila* is a rod-shaped bacterium that exhibits a negative Gram stain characteristic. This bacterium is classified as an opportunistic pathogen and is commonly found in aquatic environments and soil (Stratev and Odeyemi, 2016). The microorganism has been extensively studied because of its widespread presence in estuarine environments, food sources, and water bodies, as well as its resistance to antibiotics and ability to cause illness in both animal and human populations (Awan et al., 2018). Recent studies have brought attention to the rise of mobile strains of Aeromonas, particularly *A. hydrophila*, as the primary causative agents of different infections. Several research works have shown that *A. hydrophila* can lead to bacterial sepsis and lethal hemorrhagic consequences in a range of aquatic organisms such as *tilapia*(*Oreochromis mossambicus*), *crucian carp*(*Carassius carassius*), *northern snakehead fish* (*Channa argus*) and *zebrafish*(*Danio rerio*) (Cao et al., 2022; Li et al., 2019; Phumkhachorn and Rattanachaikunsopon, 2020; Srivastava et al., 2017). Furthermore, *A. hydrophila* has been identified in amphibian species, including *frogs*(*Rana nigromaculata*) (Xu et al., 2017). *Aeromonas hydrophila*, identified as a foodborne pathogen, has been documented to cause infections in both humans and animals on numerous occasions (Ceylan et al., 2009; Qadri et al., 1991; Roges et al., 2020; Wang et al., 2021). Despite the widespread utilization of antibiotics and ongoing initiatives to find alternatives, including Chinese herbal medicine (Rehman et al., 2016), vaccines (Frost et al., 2023), and microecological agents (Liu J. et al., 2023), there remains a significant deficiency in effective treatments against *A. hydrophila* within the entire fish culture sector. Consequently, the prevention and management of *A. hydrophila* infections continue to be a critical issue for the global aquaculture industry.

Numerous bioactive compounds derived from plants have been identified as effective agents in the prevention of diseases in fish, owing to their notable pro-growth, anti-inflammatory, antioxidant, and non-specific immunomodulatory properties. Furthermore, due to their distinctive non-toxic and environmentally sustainable characteristics, these compounds are increasingly being integrated into the aquaculture industry (Harikrishnan et al., 2021; Yu et al., 2022), Li et al. demonstrated that natural compounds, including astaxanthin, possess the ability to mitigate oxidative stress and damage to cellular barriers induced by various stimuli via the Nrf2 and GPX4 signaling pathways (Li et al., 2024). Baicalin, a flavonoid compound and the primary constituent of *Scutellaria baicalensis*, an herbal remedy in traditional Chinese medicine, exhibits diverse pharmacological properties, notably demonstrating wide-ranging antibacterial and antiviral capabilities (Li L. et al., 2023; Liao et al., 2021). Furthermore, baicalin exhibits notable anti-inflammatory characteristics, which are anticipated to mitigate the activation of the TLR4/NF-κB/P14 signaling pathway induced by lipopolysaccharides (LPS). This modulation is expected to diminish the inflammatory response associated with bacterial infections (Fu et al., 2021; Niu et al., 2024). Additional studies have corroborated the anti-inflammatory properties of baicalin. In the *carp*(*Cyprinus carpio*) gill oxidative stress test species, baicalin intervention could inhibit cell necroptosis and alleviate gill damage exposed to Chlorpyrifos (CPF, an organophosphorus pesticide) (Li X. et al., 2023). Similarly, baicalin, as one of the six anti-inflammatory drugs, showed a strong inhibitory effect on intestinal inflammation in *zebrafish* TCM test screening for inflammatory enteritis (Yu et al., 2021). It is worth noting that, in contrast to antibiotics, extracts from traditional Chinese medicine and their constituent components are typically generated through safe and efficient methods that employ mild extraction techniques (Tang et al., 2021).

As of now, there is a lack of literature regarding the potential anti-infective properties of baicalin in relation to *A. hydrophila*. The capacity of baicalin to modulate gut microbiota and mitigate organ damage resulting from *A. hydrophila* infection, as well as the specific mechanisms underlying its anti-infective effects, remain to be elucidated. Consequently, the current study aims to evaluate the effectiveness of baicalin against *A. hydrophila* and to contribute to the understanding of herbal alternatives to antibiotics within aquaculture practices.

## 2 Materials and methods

### 2.1 Bacterial culture

*A. hydrophila* strain ATCC 7966 was procured from the General Microbiological Culture Collection Center of China and preserved in a glycerol: LB medium (1:1) at −80°C. Colonies were selected and cultivated until they reached mid-log phase at a temperature of 28°C. For *in vitro* testing, *A. hydrophila* was diluted at a ratio of 1:1000 (*bacterial* solution: LB medium) and resuscitated at 28°C and 180 rpm/min until the optical density at 600 nm (OD_600_) reached 0.6 as measured by a microplate reader. *In vivo* experiments were carried out using a bacterial solution with a concentration of 2 × 10^5^ CFU/mL.

### 2.2 *In vitro* test

#### 2.2.1 The determination of the minimum inhibitory concentration (MIC)

Baicalin, verified to be 98% pure through HPLC, was procured from Shanghai McLean Biochemical Technology Co., Ltd. and stored at a temperature of 4°C. Prior to application, baicalin was mixed with LB medium to achieve final concentrations of 62.50, 31.25, 15.6, 7.8, and 3.9 mg/mL. Following this, a bacterial solution of 1.0 × 10^8^ CFU/mL was added to each well of the 96-well plate, with an additional LB solution containing bacteria serving as a control. Following a 24 h incubation period at 28°C, the turbidity of each well was evaluated, and the MIC value was determined as the lowest concentration of baicalin at which aseptic growth was visually observed.

#### 2.2.2 Effect of baicalin on the growth of *A. hydrophila*

*Aeromonas hydrophila* was diluted in sterile PBS solution to a concentration of 1 × 10^8^ CFU/mL and introduced into LB medium at a ratio of 1% (V/V). Various concentrations of baicalin, specifically 1/16 MIC, 1/8 MIC, 1/4 MIC, 1/2 MIC, and 1 MIC, were added to the culture. The mixture was then incubated in a temperature-controlled oscillator at 28°C and 180 rpm/min. The optical density at 600 nm was measured in the culture solution every 2 h, and subsequently, a growth curve was constructed.

#### 2.2.3 Effect of baicalin on the cell wall of *A. hydrophila*

*A. hydrophila* was initially diluted to a concentration of 1 × 10^8^ CFU/mL. Subsequently, baicalin was introduced to achieve final concentrations equivalent to 1 MIC, 1/2 MIC, and 1/4 MIC. With an additional LB solution containing bacteria serving as a control. The bacterial cultures were then incubated as previously outlined. At time points of 0, 2, 4, and 6 h, 3 mL of the bacterial suspension were sampled and subjected to centrifugation at 5000 rpm for 10 min. The resulting supernatant was processed in accordance with the guidelines provided by the AKP (A059-2-2) test kit from Nanjing Jiancheng Biological Company.

#### 2.2.4 Effect of baicalin on the morphology of *A. hydrophila*

The *A. hydrophila* concentration was adjusted to 1 × 10^8^ CFU/mL, and baicalin was added at a final concentration of 1/4 of the MIC. The control group received an equal amount of LB, and the bacterial solution was incubated for 8 hours. The bacterial suspension was then treated with a 2.5% glutaraldehyde solution for 2 h, followed by mixing with a 1% sodium phosphotungstate buffer. The resulting bacterial suspension was drawn up using a sterile capillary pipette and deposited onto a copper grid. After drying, the samples were examined for morphological changes in the bacteria using transmission electron microscopy.

#### 2.2.5 Effect of baicalin on *A. hydrophila* biofilm

*A. hydrophila* was cultured to the logarithmic growth stage. After collection, it was washed three times with PBS and transferred to LB medium containing 1/4 MIC, 1/2 MIC and 1 MIC baicalin at the inoculating rate of 1.0 × 10^8^ CFU/well. The culture was carried out in 24-well plates with 1 mL per well, and incubated at constant temperature at 28°C. After 72 h, the supernatant in every well was gently absorbed and cleaned with PBS to remove the floating bacteria. Fix with 500 μL 99% methanol and place it in the oven at 60°C for 30 min. Then absorb and dispose of the fixative, clean with PBS, add 1% crystal violet solution for dyeing, centrifuge at room temperature with 120 rpm for 30 min. Discard the dye solution, clean with PBS, absorb and discard the residual solution, dry in the oven at 37°C for 30 min, dissolve the crystal violet biofilm with 30% glacial acetic acid, and shake at 150 rpm for 15 min. The OD value at 570 nm for each well was determined.

#### 2.2.6. Effect of baicalin on proteins of *A. hydrophila*

Baicalin was incorporated into the bacterial solution to achieve concentrations equivalent to 1/4 MIC, 1/2 MIC, and 1 MIC, with an additional LB solution containing bacteria serving as a control. The bacteria were cultivated under these specified conditions. At intervals of 2, 4, 6, 8, and 10 h, 3 mL of the bacterial solution was extracted for centrifugation at 5000 rpm for 10 min. The resulting supernatant was decanted and analyzed using a BCA Protein Concentration Assay kit (PC0020, Beijing Solarbio Technology Co., LTD., China).

### 2.3 *In vivo* test

#### 2.3.1 Fish feeding

270 yellow catfish, with an average initial weight of (11.0 ± 0.15) g, were randomly allocated into 18 glass fiber tanks measuring 1.0 m × 0.5 m × 0.3 m, with 15 fish in each tank. The fish were provided with adaptive feeding for a period of 7 days prior to the experiment. The animal trials in this research were carried out in compliance with the Regulations on the Administration of Animal Experiments in China and received approval from the Animal Ethics Committee of Yangtze University (YJ202344).

#### 2.3.2 Challenge test

Before being exposed to *A. hydrophila*, baicalin was given to the subjects through intraperitoneal injection at varying doses of 10 mg/kg, 15 mg/kg, 25 mg/kg, and 35 mg/kg. The control and experimental groups were administered equivalent amounts of normal saline through intraperitoneal injection. 4 h later, each fish in the baicalin and experimental groups received an intraperitoneal injection of 100 μL of bacterial solution (2 × 10^5^ CFU/mL), while the control group received 100 μL of normal saline. The mortality rates of the yellow catfish were monitored for three consecutive days following the challenge.

#### 2.3.3 Sample collection

All yellow catfish were anesthetized with 100 mg/L MS-222 (Tricaine mesylate, Sigma, USA) before dissection. Collect the liver, spleen and intestine, remove the connective tissue and dry on absorbable paper, and then weigh the organs for calculating the organ index. All tissues were stored at −80°C for later use.


Organ⁢index=weight⁢of⁢organs/body⁢weight

### 2.4 Activity levels of antioxidant enzymes

The liver sample was weighed for 0.2 g, mixed with 1.8 mL of PBS and appropriate amount of tissue grinding beads, homogenized at 4°C, 60HZ, 30 s, 3 times, and then centrifuged at 3000 rpm for 10 min. The supernatant was collected, and the activities of MDA (A003-1-2), T-SOD (A001-3-2), GSH-Px (A005-1-2), CAT (A007-1-1), ACP (A060-2-2) and AKP (A059-2-2) were assessed using kits obtained from Nanjing Construction Bioengineering Institute, China.

### 2.5 qPCR validation of differentially expressed genes

RNA extraction was performed using Trizol (15596026, Thermo Fisher Scientific Inc., USA). Reverse transcription and RT-qPCR were performed using PCR (T100™, Bio-Rad Life Medical Products (Shanghai) Co., LTD, China) and Bio-rad PCR(CFX96, Bio-Rad Life Medical Products (Shanghai) Co., LTD, China) instrument following the procedures provided in the instructions of RK20428 and RK21206 (Wuhan Abclonal Biotech Co., LTD.), with *gadph* as an internal control. The expression of *cat*, *gsh*, *sod*, *keap1*, *il-1*, *ifn-γ* and *tnf-α* genes was analyzed using the SYBR Green Fast qPCR Mix (Tongsri et al., 2023). Gene-specific primer sequences for the target and reference genes were synthesized by Shanghai Sangon (Shanghai Sangon Biotechnology Co., LTD.), as shown in Table 1. The relative expression of the target gene was calculated using the 2^–△△CT^ comparative CT method (Nolan et al., 2006).

**TABLE 1:** The primers used in this study.

Gene Name	Sequence(5′-3′)	Accession numbers	Gene ID	product size (bp)
*cat*	F:CAGGAAACAACACCCCCA R:CCAAAAATCCCAAACCAT	KX455919.1	1043379042	187
*gsh*	F:TCAGGTTCCCTTGGTT R:TTTGGTCAGCAGGTGTA	XM_047807258.1	2227620442	223
*gsh*	F:TTGGAGACAATACAAATGGGTG R:CATCGGAATCGGCAGTCA	KX455916.1	1043379036	209
*ifn-γ*	F:TACAGAGCGAAGAACAAC R:CCTGAGCCAGAAACCT	XM_027151670.2	2227598702	197
*il-1*	F:CAGCGACTGTGGATTTGAC R:TGGATACGGATTCCTTTGT	XM_027139384.2	2227608016	253
*keap1*	F:CTACGCCCTTCCACCCAC R:GCCCGAACAAAACACACG	XM_047813316.1	2227644005	228
*tnf-α*	F:GTGTCGGGGGAGTTTATC R:ACCTTCTTCGTTTGGCTT	JAJLOY010000004.1	113655347	291
*gapdh*	F:TCGGCATCAACGGATTTGGC R:AGCCTTGACCTCTCCCTTGT	KP938521.1	113644399	275

*cat*: The enzyme catalase, *gsh*: Glutathione peroxidase, *sod*: Superoxide dismutase, *ifn-γ*: Interferon-gamma, *il-1*: Interleukin-1, *keap1*: Kelch-like ECH-associated protein-1, *tnf-α*: Tumor necrosis factor-alpha, *gapdh*: Glyceraldehyde-3-phosphate dehydrogenase.

### 2.6 Observation of tissue pathology

Liver and intestinal tissues were preserved in 4% paraformaldehyde, dehydrated using a range of alcohol concentrations, cleared with xylene, embedded, serially sectioned and stained with HE, and finally sealed with neutral gummy, visualized and captured using an XD30A-RFL microscope (Ningbo Sunshine Optoelectronic Information Co., LTD, China).

### 2.7 16s RNA sequencing of intestinal contents

SDS methods was employed for the extraction of DNA from the samples, and agarose gel electrophoresis was utilized to assess the purity and concentration of the extracted DNA. Subsequently, an appropriate amount of DNA was taken into a centrifuge tube and diluted to 1 ng/μL with sterile water. PCR amplification was performed using primers for the 16S V4 region (515F and 806R). The PCR products were detected by electrophoresis in 2% agarose gel, and the samples were mixed in equal quantities according to the concentration of PCR products. After fully mixing, the samples were detected again by electrophoresis in 2% agarose gel. The target bands were recovered by gel recovery kit provided by Qiagen company. According to the standard operating procedure of PCR library construction, the purified amplification fragments were constructed into Illumina library, and the PE300 sequencing was performed on the Illumina Miseq platform. The sequence data underwent demultiplexing, quality filtering, and analysis using QIIME and R Language (v4.2.0). Principal coordinates analysis (PCoA) was conducted based on bray-curtis matrices, with statistical significance determined by permutational multivariate analysis of variance (PERMANOVA) to assess differences in beta diversity between groups. The analysis of differential abundant taxa across groups was performed using Linear discriminant analysis effect size (LEfSe) with default parameters. Venn analysis was used to identify shared and unique species across different groups. Nonmetric Multidimensional Scaling analysis (NMDS) was used to analyze the structure of microbial communities in different groups. General statistical analysis and visualization of results were carried out using R (version 4.1.3) and packages including vegan (v2.6-4), phyloseq (v1.38.0), tidyverse (v1.3.2), ggpubr (v0.5.0), ComplexHeatmap (v2.10.0) and corrplot (v0.92).

### 2.8 Statistical analysis

Statistical analyses were performed with SPSS 25.0 (Chicago, United States). One-way analyses of variance (ANOVA) was performed on the data. Data were presented as mean ± SD (standard deviation). Tukey’s test was used to compare the mean values of treatments when overall differences appeared significant (*P* < 0.05). The results were plotted using Graphpad prism 8.0.

## 3 Results

### 3.1 Antibacterial evaluation of baicalin against *A. hydrophila in vitro*

Following a 24 h incubation, it was observed that at concentrations of 62.50, 31.25, 15.60, and 7.80 mg/mL of baicalin, the growth of *A. hydrophila* ceased and the culture medium remained transparent. The MIC of baicalin against *A. hydrophila* was determined to be 7.80 mg/mL, with the inhibitory effect exhibiting a direct correlation with the dosage administered.

### 3.2 Effect of baicalin on the proliferation and cellular structure of *A. hydrophila*

**FIGURE 1:**
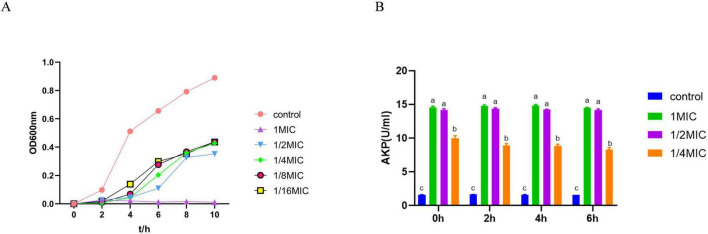
The effect of baicalin on the growth curve and the release of AKP of *A. hydrophila*, **(A)** the abscissa is the time hour, and the ordinate is the absorbance at OD600nm. **(B)** the abscissa is the time, and the ordinate is the enzyme activity unit. All experiments were repeated more than three times and presented as mean ± SD, significant differences (*P* < 0.05) between groups with different superscripts a, b, c, and d.

The effect of baicalin on the growth of *A. hydrophila* was demonstrated in Figure 1A. The logarithmic growth phase of *A. hydrophila* in the control group was at the stage of 0–2 h, and the stable growth phase was at the stage of 4–10 h. Baicalin at the concentration of 1/2 MIC delayed the time of entering the stable phase, and had a certain inhibitory effect on the growth of the strain. The growth of *A. hydrophila* was obviously inhibited by baicalin at the concentration of 1 MIC.

Figure 1B depicted the changes in AKP content. The AKP content in the 1 MIC, 1/2 MIC, and 1/4 MIC groups showed a notable increase compared to the control group (*P* < 0.05), indicating that baicalin may have the ability to interfere with the cell wall of *A. hydrophila.*

### 3.3 Effects of baicalin on morphology, biofilm, and proteins of *A. hydrophila*

**FIGURE 2:**
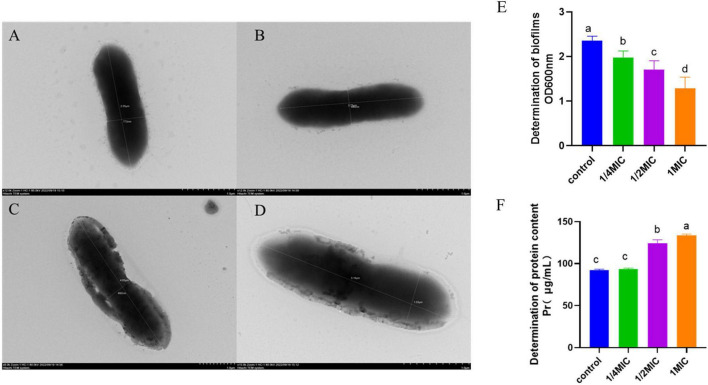
Effects of baicalin on *A. hydrophila* morphology, biofilm, and protein, **(A,B)** *A. hydrophila* morphology in control group, **(C,D)** *A. hydrophila* morphology after baicalin treatment. **(E)** effect of baicalin on biofilm formation of *A. hydrophila*, **(F)** effect of baicalin on protein release of *A. hydrophila*. All experiments were repeated more than three times and presented as mean ± SD, significant differences (*P* < 0.05) between groups with different superscripts a, b, c, and d.

The morphology of *A. hydrophila* was depicted in Figures 2A-D. In comparison to the control group (Figures 2A, B), at a concentration of 1/4 MIC of baicalin (Figures 2C, D), the bacteria displayed a rough appearance and evident structural damage, including a broken bacterial wall. The findings of the biofilm analysis are presented in Figure 2E. It was observed that the presence of *A. hydrophila* biofilm was notably suppressed following various concentrations of baicalin treatment in comparison to the control group (*P* < 0.05). Figure 2F illustrates alterations in extracellular protein levels subsequent to baicalin administration. While no significant variance was noted between the 1/4 minimum inhibitory concentration (MIC) groups and the control group (*P* > 0.05), a substantial rise in extracellular protein content was evident in a dose-dependent manner with increasing baicalin concentrations (*P* < 0.05, *P* < 0.001).

### 3.4 Survival of challenged fish

**FIGURE 3:**
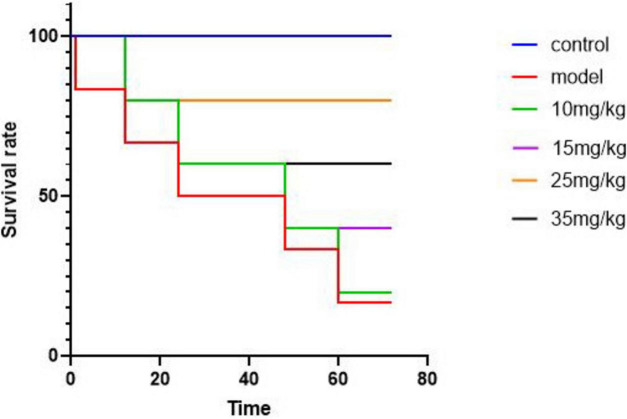
Survival curves of yellow catfish infected with *A. hydrophila* by baicalin, abscissa denotes time and ordinate denotes survival.

The Figure 3 illustrates the survival rates of yellow catfish under different experimental conditions. Following a challenge test, mortality was observed in both the model group and the groups treated with baicalin after 12 hours. By the 24-hour mark, the model group exhibited a mortality rate of 50%. However, by 72 hours post-challenge, the mortality rates in the baicalin groups administered with 10 mg/kg and 15 mg/kg had decreased. Notably, the group treated with 25 mg/kg of baicalin displayed the lowest mortality rate at 25%.

### 3.5 Organ index

The organ indexes of yellow catfish after *A. hydrophila* infection and baicalin treatment were shown in Table 2. Compared with the control group, the liver index in the model group increased significantly (*P* < 0.05), while decreased significantly (*P* < 0.05) after injection of baicalin. The spleen index in the model group showed a significant decrease compared with the control group (*P* < 0.01), while compared with the model group, the spleen index of the different concentrations of baicalin groups was significantly increased (*P* < 0.05), although the 25 mg/kg baicalin group showed significant difference (*P* > 0.05), but still showed an upward trend. Compared with the control group, the intestinal index of the model group was significantly decreased (*P* < 0.05), and after baicalin intervention, the intestinal index of each group returned to normal level.

**TABLE 2:** Organ indexes of yellow catfish after *A. hydrophila* infection and baicalin intervention.

Organ index	Control	Model	10 mg/kg	15 mg/kg	25 mg/kg	35 mg/kg
Liver	1.18 ± 0.19b	2.58 ± 0.29a	1.49 ± 0.30b	1.41 ± 0.31b	1.44 ± 0.18b	1.28 ± 0.11b
Spleen	0.33 ± 0.03a	0.13 ± 0.03c	0.32 ± 0.07ab	0.28 ± 0.09ab	0.23 ± 0.08bc	0.28 ± 0.05ab
Intestine	1.61 ± 0.26a	0.66 ± 0.21c	1.12 ± 0.37b	1.32 ± 0.18ab	1.26 ± 0.15ab	1.34 ± 0.56ab

All experiences were repeated three times and expressed as mean ± SD, significant differences between groups with different superscripts a, b, c, and d (*P* < 0.05).

### 3.6 Antioxidant parameters of liver

**FIGURE 4:**
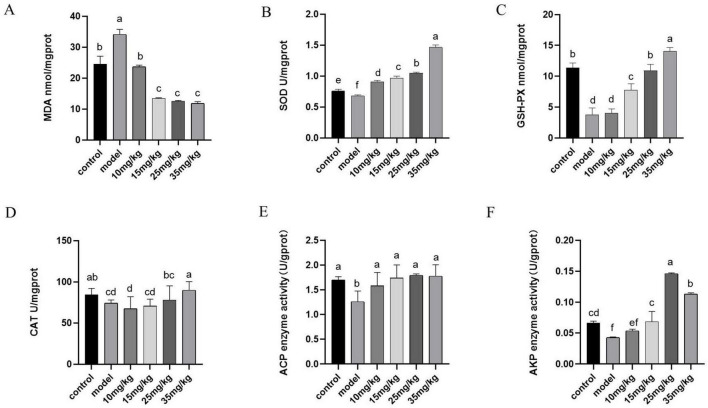
Effects of baicalin on antioxidant capacity and immunity of yellow catfish liver infected by *A. hydrophila*. **(A)** MDA expression level in liver, **(B)** SOD expression level in liver, **(C)** GSH-Px expression level in liver, **(D)** CAT expression level in liver, **(E)** ACP expression level in liver, **(F)** AKP expression levels in the liver. All experiments were repeated more than three times, and all were expressed as mean ± SD. The differences between groups with different superscripts a, b, c and d were significant (*P* < 0.05).

Figure 4 showed the antioxidant results of yellow catfish after *A. hydrophila* infection and baicalin intervention. It was evident that the model group exhibited a notable increase in MDA expression level compared with the control group (*P* < 0.05), but decreased significantly after baicalin treatment (*P* < 0.05). Furthermore, the model group showed significantly lower expression levels of SOD, GSH-Px, CAT, ACP and AKP compared with the control group (*P* < 0.05). Following baicalin treatment, there was a significant dose-dependent increase in the expression levels of these indicators (*P* < 0.05, *P* < 0.01).

### 3.7 Results of RT-PCR

**FIGURE 5:**
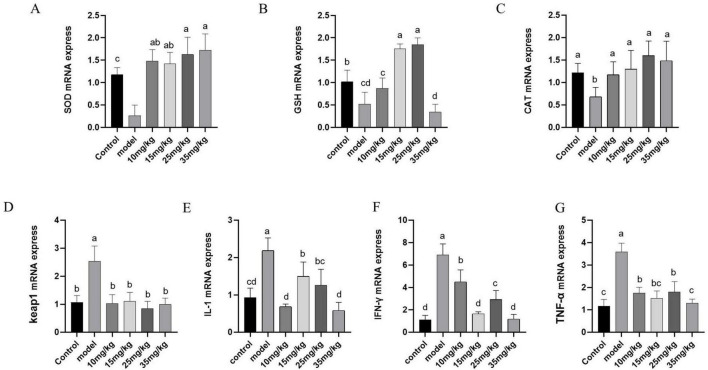
Effect of baicalin on mRNA expression of antioxidant and immune-related genes in the spleen of yellow catfish infected with *A. hydrophila*. **(A)** *sod* mRNA expression; **(B)** *gsh* mRNA expression; **(C)** *cat* mRNA expression; **(D)** *keap1* mRNA expression; **(E)** *il-1* mRNA expression; **(F)** *ifn-γ* mRNA expression; **(G)** *tnf-α* mRNA expression. All experiences were repeated more than three times and presented as mean ± SD. Significant differences between groups with different superscripts a, b, c, and d (*P* < 0.05).

The mRNA expressions of *sod, gsh* and *cat* in the spleen were shown in Figure 5. The levels of *sod*, *gsh* and *cat* mRNA expressions in the model group were significantly lower than those in the control group (*P* < 0.05). Baicalin supplementation at different doses significantly increased the mRNA expression levels of *sod* and *cat* (*P* < 0.01), and the increased effects were in a dose-dependent manner. While, the mRNA expression level of *gsh* was up-regulated (*P* > 0.05) in baicalin supplementation groups, but there was no significant differences compared with that in the model group. The mRNA expression levels of *keap1*, *il-1*, *ifn-γ* and *tnf-α* in spleen were shown in Figures 5D–G. Compared with the control group, these indexes were significantly increased after *A. hydrophila* challenge (*P* < 0.01). After baicalin supplementation, the expression levels of these indicators were significantly decreased (*P* < 0.01).

### 3.8 Results of histopathology

**FIGURE 6:**
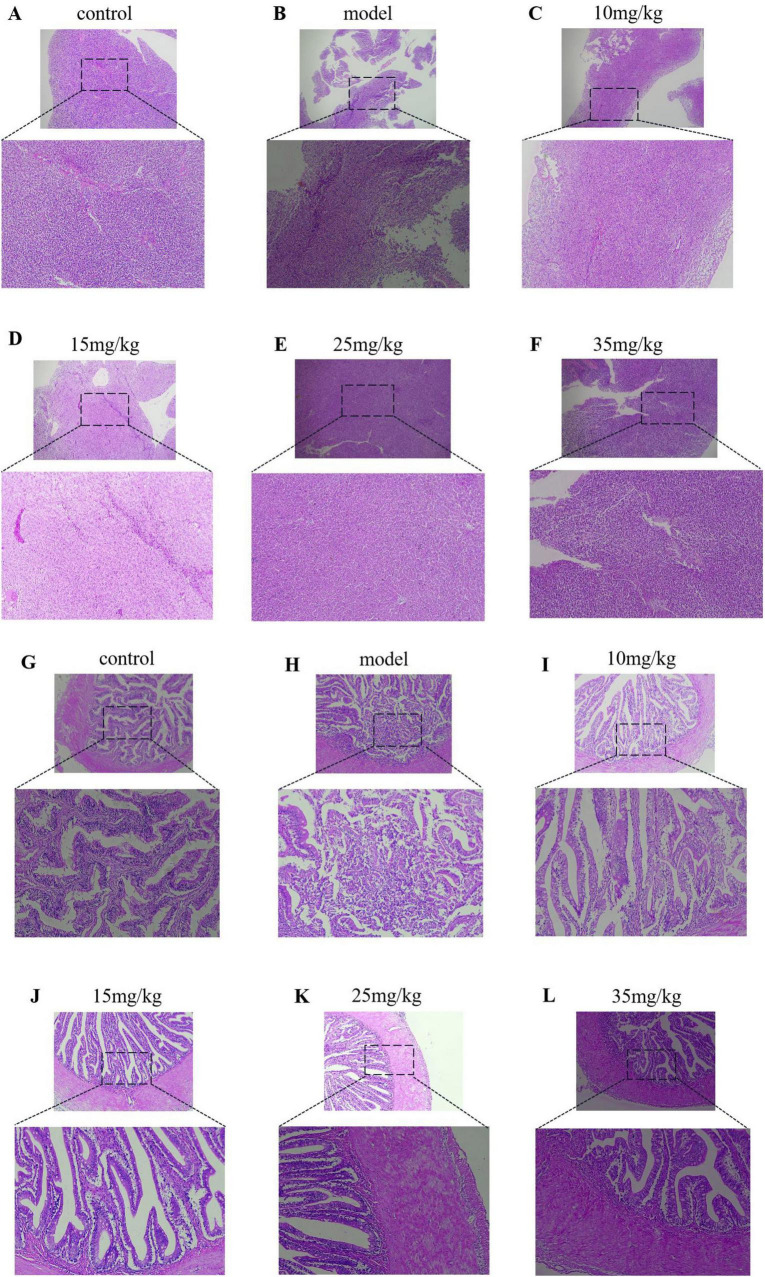
Results of HE staining of the liver and intestine of yellow catfish injected with baicalin and infected with *A. hydrophila*, **(A–F)** HE staining of liver tissue pathology, **(G–L)** HE staining of intestinal tissue pathology.

The histopathological results, as shown in Figure 6, showed that the liver structure was intact in both the control and baicalin treated groups (Figures 6A, C–F), and the hepatocytes were neatly arranged and clearly outlined, without obvious pathological manifestations. In the model group, the liver lesions were obvious, including large area necrosis, structural homogenization, extensive granular degeneration, disordered cell arrangement, and a large number of inflammatory cells infiltration (Figure 6B). The relative quantitative findings are presented in Supplementary Table 1. In comparison to the control group, the model group exhibited an inflammatory cell infiltration rate of 48.6%, which was statistically significant (*P* < 0.05). In comparison to the model group, Administration of varying doses of baicalin (10 mg/kg, 15 mg/kg, 25 mg/kg, and 35 mg/kg) resulted in a reduction of inflammatory cell infiltration to 44.62%, 35.84%, 36.10%, and 36.10%, respectively, with these differences also being statistically significant (*P* < 0.05).

In pathological sections of the intestine, the intestinal villi in the model group were disrupted and a large number of lymphocytes and neutrophils were infiltrated (Figure 6G). There was a small amount of inflammatory cell infiltration in the intestinal tissue of baicalin 10 mg/kg group (Figure 6H), while the intestinal tissue integrity of baicalin 15 mg/kg group was significantly improved compared with that of baicalin 10 mg/kg group (Figure 6I). The intestinal tissues of baicalin 25 mg/kg and 35 mg/kg had clear boundaries and intact structures without obvious pathological manifestations (Figures 6J, K). The relative quantitative analysis indicated that the length of intestinal villi in the model group (225.40 μm) was significantly reduced compared to the control group (276.23 μm), with a statistically significant difference observed (*P* < 0.05). Administration of Baicalin at various dosages (10 mg/kg, 15 mg/kg, 25 mg/kg, and 35 mg/kg) resulted in an increase in the length of intestinal villi affected by *A. hydrophila*, measuring 305.50 μm, 308 μm, 327 μm, and 340.75 μm, respectively. With a statistically significant difference observed (*P* < 0.05). These results indicate that baicalin ina certain concentration range can improve the pathological changes of liver and intestinal tissue yellow catfish infected by *A. hydrophila*.

### 3.9 Analysis of intestinal flora

#### 3.9.1 Analysis of microbiota composition

**FIGURE 7:**
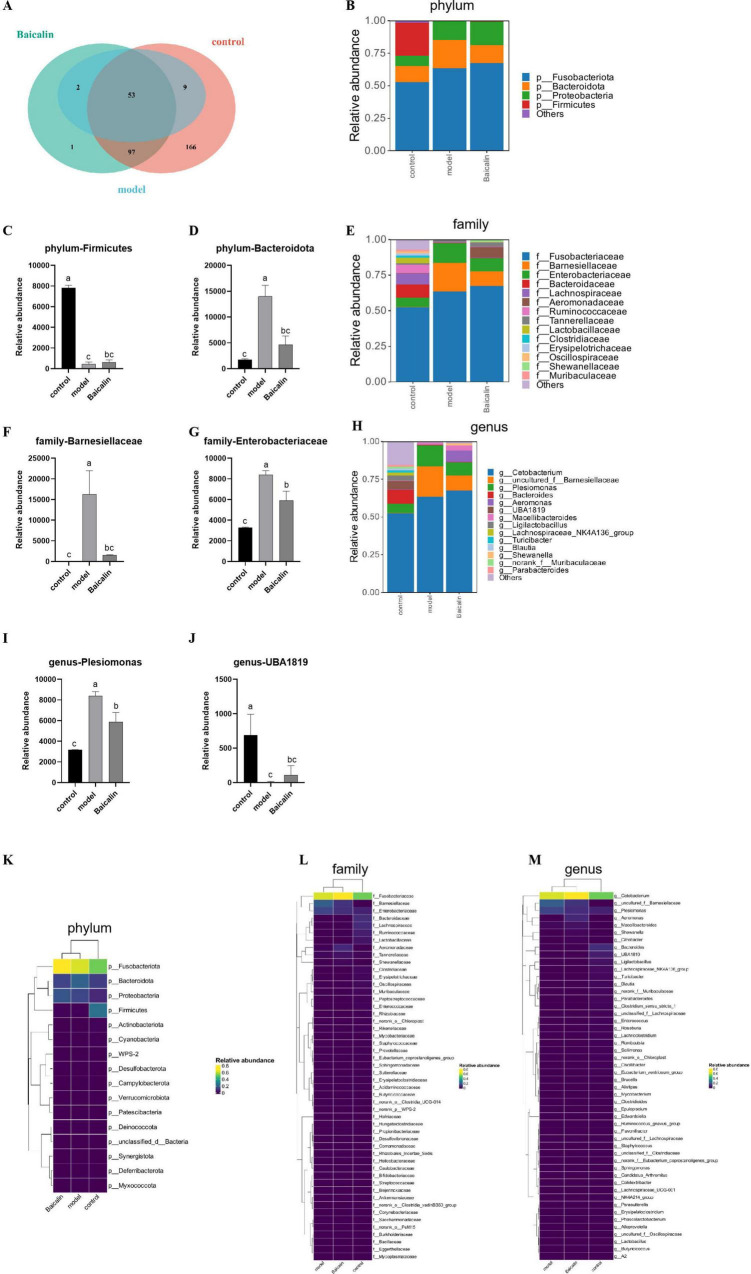
Effect of baicalin on intestinal microbial composition of yellow catfish infected with *A. hydrophila.* **(A)** Venn diagram of intestinal otus of yellow catfish under three treatment conditions. The non-overlapping part of the diagram is unique to each group, and the overlapping part is common to each group, and the numbers are marked in the corresponding range, **(B)** Distribution of flora at Phylum level, **(C)** Relative abundance of *Firmictues* in different groups, **(D)** Relative abundance of *Bacteroidota* in different groups, **(E)** Distribution of flora at family level, **(F)** Relative abundance of *Barnesiellaceae* in different groups, **(G)** Relative abundance of *Enterobacteriaceae* in different groups, **(H)** Distribution of bacteria at genus level, **(I)** relative abundance of *Plesiomonas* in different groups, **(J)** relative abundance of UBA1819 in different groups, **(K)** Clustering heat map of different bacteria at Phylum level, **(L)** Heatmap of differential flora at family level, **(M)** Heatmap of differential flora at genus level. All test samples were repeated three times, and different letters a, b, c and d indicated significant differences (*P* < 0.05).

Venn diagram of gut microbiota composition can show the number of common and different OTUs among samples under different conditions, which can further evaluate the diversity and similarity of microbiota between groups. As shown in Figure 7A, the total number of OTUs was 325, 153 and 64 in the control group, baicalin group and model group, respectively.

As shown in Figures 7B, E, H, The intestinal flora of catfish is composed Fusobacteriota, Bacteroidota, Proteobacteria and Firmicutes at the phylum level. Compared with the control group, there was a notable decrease in the abundance of Firmicutes in the model group(*P* < 0.05), and after baicalin supplementation, the abundance of Firmicutes increased, but the difference was not significant (*P* > 0.05) (Figure 7C). Compared with the control group, the abundance of Bacteroidota was significantly increased in the model group (*P* < 0.05), but there was no significant difference compared with the baicalin group (*P* > 0.05) (Figure 7D). At the family level, The gut microbiota predominantly consists of five microbial species, namely Fusobacteriaceae, Barnesiellaceae, Enterobacteriaceae, Bacteroidaceae, and Lachnospiraceae. The abundance of Barnesiellaceae and Enterobacteriaceae was significantly increased in the model group (*P* < 0.05) (Figures 7F, G), while there was no significant difference in Barnesiellaceae between the baicalin group and the control group (*P* > 0.05). It was worth noting that although the number of Enterobacteriaceae in the baicalin group was significant lower than that in the model group (*P* < 0.05). Similarly, at the genus level, 5 species were *Cetobacterium*, *uncultured_f_Barnesiellaceae*, *Plesiomonas*, *Bacteroides* and *Aeromonas*. Compared with the control group, the abundance of *Plesiomonas* was significantly increased in the model group (*P* < 0.05), but decreased after baicalin supplementation (*P* < 0.05) (Figure 7I). The abundance of *UBA1819* was significantly decreased in the model group compared with the control group (*P* < 0.05), but increased after baicalin supplementation (*P* < 0.05) (Figure 7J).

In order to analyse the relative abundance of other microorganisms and their distribution in groups, cluster heat maps were used to show the distribution of bacteria at plyum, family and genus levels in Figures 7K–M.

#### 3.9.2 Analysis of microbial α and β diversity

**FIGURE 8:**
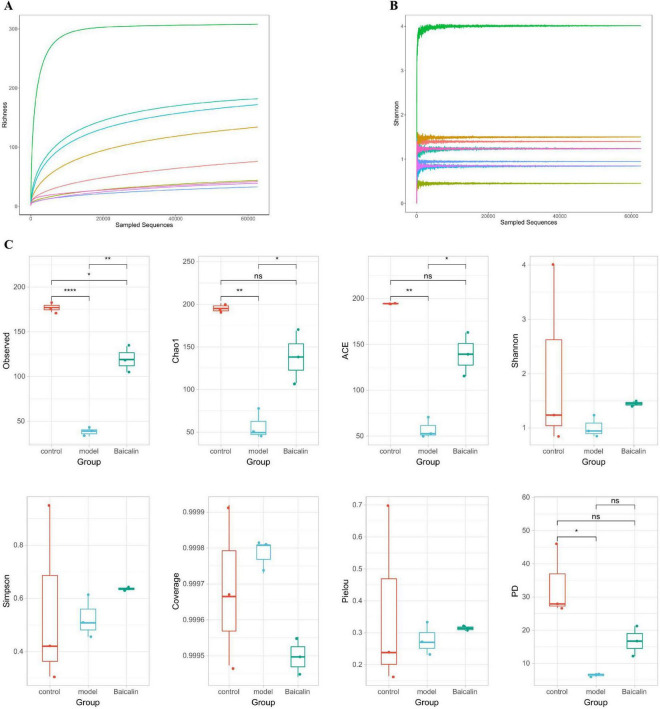
Analysis of intestinal microbial alpha diversity in yellow catfish infected with *A. hydrophila* by baicalin. **(A)** sample dilution curve, the abscissa represents the amount of randomly selected sequencing data, and the ordinate represents the number of observed taxa, **(B)** Shannon-Wiener curve, abscissa is sequencing depth, ordinate is Shannon index, **(C)** Alpha diversity index, with the abscissa representing sample grouping and the ordinate representing diversity index values. If there is a significant difference (*P* < 0.05, *P* < 0.01), a significant marker is displayed between groups, usually denoted by “* or **”, otherwise denoted by “ns”. ****P < 0.001.

Dilution curves are utilized to compare the diversity of species in samples with varying levels of sequencing data, as well as to assess the adequacy of sequencing data for a given sample. Additionally, the Shannon-Wiener is employed to construct curves representing the microbial diversity index at different sequencing depths for each sample. The sample dilution curves and Shannon curves shown in Figures 8A, B indicated that the cumulative curves of the nine sample species gradually flattened and the sequencing data were sufficient to support subsequent analyses.

The OTU characteristic sequences obtained by Alpha diversity analysis were analyzed for differences in species diversity between groups using Tukey and Kruskal-Wallis rank sum tests. Observed (Figure 8C), Chao1 ACE and PD in the model group were significantly different from those in the control group (*P* < 0.05, *P* < 0.01), and the Observed index of baicalin group was significantly different (*P* < 0.05). There were significant differences in Observed, Chao1 and ACE in baicalin group (*P* < 0.05).

**FIGURE 9:**
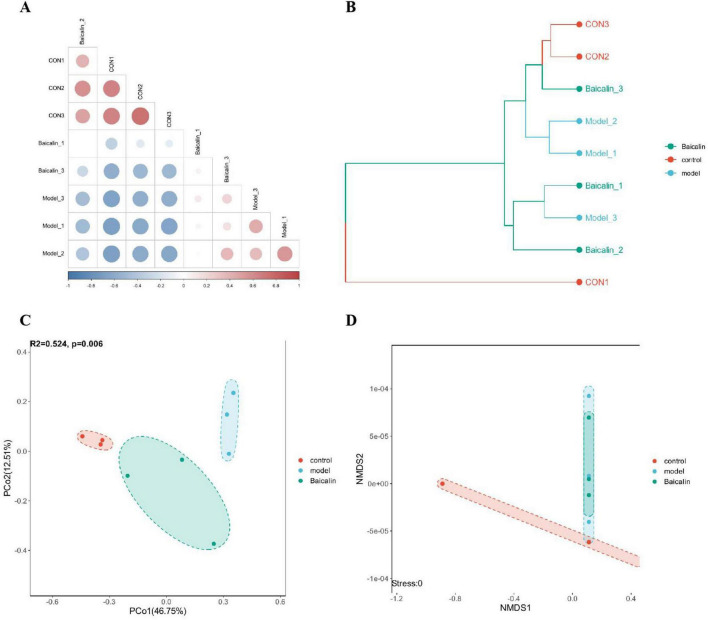
Analysis of intestinal microbial beta diversity in yellow catfish infected with *A. hydrophila* by baicalin. **(A)** Heatmap of sample correlation matrix. The closer the samples are, the closer the color is to red. The larger the sample difference is, the closer the color is to blue, **(B)** sample hierarchical clustering analysis, branch length represents the distance between samples, and samples are distinguished by different colors, **(C)** PCA analysis, points in the figure represent samples, and different colors/shapes represent different grouping information, **(D)** NMDS analysis, points in the NMDS graph represent samples, different colors/shapes represent different grouping information, and the distance between the lines of each sample point reflects the similarity between each sample, the shorter the distance, the greater the similarity. The distance of sample points in the same group indicates the repeatability of samples, and the distance of samples in different groups reflects the difference in rank (data ranking) of sample distances between groups.

The obtained OTU characteristic sequences were analyzed for Beta diversity, and the coefficient of difference between samples was measured using unweighted unimodal distance, as shown in Figure 9A. The hierarchical clustering algorithm was used to calculate the difference among the three groups of samples, as shown in Figure 9B. The proximity of the samples was positively correlated with their degree of similarity. According to the results of principal coordinate analysis (PCoA) (Figure 9C), the first two principal axes explained 46.75% and 12.51% of the sample variance data, and the three groups of samples were clearly separated. Similarly, non-metric multidimensional scaling analysis (NMDS) was used to analyze and classify the samples in multidimensional space, and the spatial localization map of the samples was finally obtained (Figure 9D).

#### 3.9.3 Species difference analysis

**FIGURE 10:**
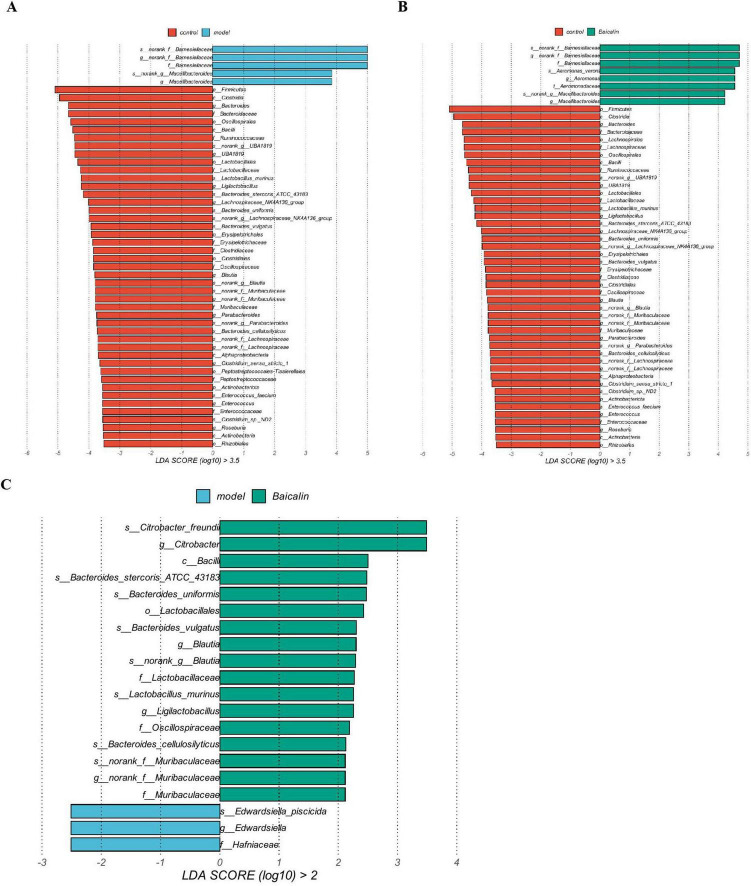
Species difference analysis. **(A)** Differential biomarkers between the control and model groups, **(B)** Differential biomarkers between control and baicalin groups, **(C)** Differential biomarkers between the model and baicalin groups. The bar chart shows the significant difference Biomaker with LDA score greater than the preset value, that is, Biomaker with statistically significant difference, the default preset value is 2.0. The color of the bar graph represents the respective group, and the length represents the LDA score, which is the degree of influence of significantly different species between groups.

The results compared the biological markers of microbial differences in the three groups of samples were shown in Figure 10. The non-parametric Kruskal-Wallis rank sum test was used to detect the biological characteristics with significant differences among the three groups. The Wilcoxon rank-sum test was used to check whether significantly different biologic features converged to the same category (if any) across subgroups. Finally, linear discriminant analysis (LDA) was used to reduce the dimensionality of the data and evaluate the influence of the significant differences in biological characteristics (LDA score). As shown in Figure 10, Barnesiellaceae and Macellibacteroides at the family level and genus level in the model group belonged to the dominant flora. While in the baicalin group, except for the above two flora, the dominant flora also included Aeromonas_veronii at the species level and Aeromonas at the genus level. Compared with the model group, baicalin may resist *A. hydrophila* infection by increasing the abundance of *Citrobacter_freundi* at species level and Citrobacter at genus level.

## 4 Discussion

Chinese herbal medicine has gradually entered the field of farming industry due to its green, safe and sustainable development characteristics under the general environment of prohibiting resistance (Wang et al., 2022). In recent years, the utilization of herbs within the aquaculture sector has gained significant traction due to their efficacy as immune enhancers. These botanical agents are recognized for their ability to facilitate growth, enhance immune responses, and improve resilience against various diseases. This approach presents a beneficial strategy for mitigating the emergence of bacterial resistance, a phenomenon that poses risks to both human health and environmental integrity as a consequence of excessive antibiotic use. Chinese herbal medicine emerges as a promising alternative to antibiotics in the protection of aquaculture from pathogenic infections, providing an effective and environmentally sustainable solution. Existing research suggests that baicalin exhibits inhibitory properties against a range of bacterial strains; however, there is a paucity of data regarding its specific effects on *A. hydrophila* (Wu et al., 2020). The research examined the immune response and resistance to pathogens in yellow catfish following an intraperitoneal injection of baicalin.

It must be acknowledged that the suppression of bacterial growth represents the most effective strategy for controlling bacterial populations (Gatadi et al., 2019). Prior to the experiment, an *in-vitro* study was conducted to check the efficiency of baicalin to inhibit bacterial growth. The results showed that baicalin at 1/2 MIC and 1 MIC concentrations not only limited the growth of *A. hydrophila* but also delayed its entry into the growth phase. The enzyme alkaline phosphatase (AKP), situated between the bacterial cell wall and cell membrane, serves as a valuable indicator for assessing cell wall integrity (He et al., 2018). The results of this investigation demonstrate that an optimal concentration of baicalin significantly increases the levels of AKP. This suggests that baicalin may partially disrupt the integrity of the cell wall in *A. hydrophila*, potentially enhancing its antibacterial or bactericidal properties. This conclusion is further corroborated by observations made through transmission electron microscopy. Generally, biofilms represent a collective growth of microorganisms, which significantly contribute to their pathogenicity and resistance against host immune responses (Chen and Wen, 2011). Therefore, the release of biofilms is one of the indicators of bacterial viability, which can be regarded as the destruction of bacteria. At this time, a large number of intracellular proteins are released, resulting in the death of bacteria (Stoodley et al., 2002). The results from the biofilm test showed that baicalin successfully decreased the formation of *A. hydrophila* biofilm by inhibiting its growth, leading to a significant protein leakage. In this study, baicalin at a concentration of 7.80 mg/mL was found to have both antibacterial and bactericidal effects. This suggests that baicalin can disrupt the extracellular wall of *A. hydrophila* and release proteins, resulting in antibacterial and bactericidal actions (Zhang et al., 2020).

Crude extract and flavonoid components of Baicalein can prevent or reduce the harmful toxic effects of the compounds (Li X. et al., 2023). Jia et al. reported that dietary supplementation of baicalein can significantly increase the growth rate of tilapia and effectively alleviate H2O2-induced liver injury (Jia et al., 2021). In this study, this findings indicated that administration of 25 mg/kg of baicalin significantly enhanced the survival rate of yellow catfish, suggesting that baicalin exerts a protective effect against *A. hydrophila* infection in this species. The liver plays a significant role in the immune surveillance of pathogens that enter the gastrointestinal tract and is also influenced by the mucosal immune response (Trivedi and Adams, 2016). Damage to both the liver and intestine can frequently facilitate the systemic dissemination of bacteria in animal models (Spadoni et al., 2015). In this study, the liver index of yellow catfish subjected to *A. hydrophila* challenge was elevated, which further corroborates the occurrence of liver injury in these fish. However, following baicalin treatment, the liver index progressively normalized. From another angle, the study observed a decreasing trend in both spleen and intestinal indexes post-infection with *A. hydrophila*, which subsequently increased following baicalin intervention. In a related study, Rivas et al. reported a significant reduction in spleen size in rainbow trout exposed to environments contaminated with oil sands and *A. hydrophila* (Rivas-Aravena et al., 2019). Similarly, a study on sturgeon infected by *A. hydrophila* showed that bacterial attack caused intestinal villi destruction, intestinal rupture, shrinkage and dehydration (Xu et al., 2023). In the present study, baicalin supplementation could recover the organ indexes of yellow catfish infected with *A. hydrophila*.

During bacterial infections, the release of inflammatory mediators such as *il-1* and *tnf-α* frequently exacerbates the oxidative stress response (Liu A. et al., 2023). The antioxidant enzyme system serves as the primary line of defense against oxidative damage, and even minor fluctuations in enzyme activity can disrupt the balance of this system. Concurrently, the presence of free radicals can facilitate macrophage migration and trigger the secretion of inflammatory and pro-fibrotic cytokines. This cascade further promotes the production of ROS and the accumulation of malondialdehyde (MDA) within cells (Atli et al., 2016). Moreover, excessive levels of ROS can enhance the expression of pro-inflammatory cytokines by activating the NF-κB (Cheng et al., 2023), Consequently, Oxidation and inflammation are interrelated processes that play a vital role in the defense mechanisms of fish against pathogenic infections (Towler, 2008). This study investigated the potential immune benefits of baicalin in yellow catfish by assessing the levels of SOD, GSH, ACP and AKP in the liver. However, after *A. hydrophila* infection, proinflammatory cytokines such as *il-1*, *ifn-γ* and *tnf-α* were detected to be up-regulated. The results indicated that *A. hydrophila* was able to cause immune stress in yellow catfish. But baicalin reduced the expression level of *il-1*, *ifn-γ* and *tnf-α* mRNA, revealed a modulating impact of baicalin on the anti-inflammatory biomarkers. In a similar vein, the research conducted by Wu et al. revealed a significant increase in the expression of inflammation-related cytokines, including *IFN-γ* and *IL-1*, in the liver and intestine of tilapia during the inflammatory response. This finding aligns with the results of the current study (Wu et al., 2016).

Inflammation is a component of the intricate pathological process in numerous diseases, resulting in tissue damage. Histopathology is a classic approach for the drug therapy efficiency evaluation (Esposito-Fava et al., 2024). In the current study, large area of liver tissue necrosis, inflammatory cell infiltration, disordered arrangement of liver cells and the destroyed intestinal villi in the model group confirmed tissues injury induced by *A. hydrophila*. However, baicalin treatment (especially in 25mg/kg) significantly attenuated inflammatory damage. These results were consistent with the research on liver inflammation in poultry induced by LPS (Cheng et al., 2017). Baicalin has the potential to protect tissues from damage caused by bacterial infection and act as a stress-reducing agent in fish.

As an essential digesting tissue for fish, gut is the most abundant organ for fish microbes, which can promote nutrient absorption, enhance immune and anti-infection ability (Deng et al., 2022; Muller et al., 2020; Zhou et al., 2018). These functions are closely associated with the diversity of intestinal microbiota; a high level of diversity, along with the presence of beneficial bacteria, enhances nutrient absorption in the gastrointestinal tract and bolsters its inherent immune response (Mohr et al., 2020). This research examined the changes of the yellow catfish’s gut microbiota after being infected with *A. hydrophila*, and the protective impact of baicalin on the yellow catfish’s gut. The study found that *A. hydrophila* infection significantly reduced the quantity and diversity of microorganisms in the yellow catfish’s intestine. Baicalin has demonstrated the capacity to enhance the innate immune response in yellow catfish through the downregulation of *Keap1*, *IL-1*, *IFN-γ*, and *TNF-α* expression. In a related investigation conducted by Zhang et al. on the inflammatory response in FHM cells induced by oxidized fish oil, exogenous L-carnitine was similarly observed to decrease the mRNA levels of Keap1 and diminish the expression of IL-1β and TNF-α (Zhang D. M. et al., 2019). This finding suggests an augmentation of innate immunity, which aligns with the results of the current study. Additionally, it may mitigate the detrimental effects of *A. hydrophila* on yellow catfish., resulting in a higher quantity and diversity of microorganisms in their gut than in control fish infected with the same dose of *A. hydrophila*.

Wu et al. identified Firmicutes and Bacteroidetes as fundamental components of the intestinal microbiota in yellow catfish (Wu et al., 2012). The findings of the present study align with these results. But the abundance of *Firmicutes* was significantly down-regulated after *A. hydrophila* infection, which may be related to the destruction of the intestinal tract of yellow catfish. This result was in agreement with the report of Naaber (Naaber et al., 1998). Firmicutes is one of the cornerstone of intestinal flora of humans and animals and belongs to beneficial bacteria, which is closely related to body obesity and health (Jandhyala et al., 2015; Lanza et al., 2015). Bacteroidota are helpful bacteria that breakdown proteins and carbohydrates (Kaakoush, 2015; Zhang W. et al., 2019). It can influence intestinal and parenteral effects to regulate homeostasis by modulating physiological functions such as metabolism, enteritis, hematopoiesis and barrier stability (Ahlawat et al., 2021). In the present study, Bacteroidetes showed no significant difference between the control and baicalin groups, but there was a trend of up-regulation in the model group infected with *A. hydrophila*, which may be related to the cause of intestinal flora imbalance (Shi et al., 2017). Research suggests that the increased presence of *Barnesiellaceae* may result in oxidative stress in fish, interfere with normal intestinal metabolism, and cause intestinal damage (Zheng et al., 2022). The family *Enterobacteriaceae*, which comprises hepato-intestinal bacteria, has been associated with the onset of Edwardiasis in fish, as evidenced by the research conducted by Leung et al. Additionally, changes in the composition of these bacterial populations can considerably compromise the immune system of fish, consequently obstructing their optimal growth (Leung et al., 2019). *Plesiomonas*, identified as a pathogen affecting fish, has been demonstrated by Liu et al. to cause damage to multiple tissues, including the heart, intestine, liver, spleen, and kidneys (Liu et al., 2015). In the current study, we observed a significant increase in the abundance of three specific bacterial groups following infection with *A. hydrophila*, which was associated with varying degrees of damage to the liver and intestine. This finding not only suggests a disruption in intestinal flora but may also be indicative of an intestinal infection. The abundance of *UBA1819*, a member of Firmicutes, was significantly down-regulated in the model group, but partially restored in the baicalin group, which may also be closely related to the function of baicalin in regulating intestinal flora. The above results are consistent with the previous findings, baicalin supplementation had significant effects on gut microbial alpha and beta diversity (Liu et al., 2020; Peng et al., 2021). In summary, baicalin was able to improve the gut microbiota imbalance and maintain homeostasis of yellow catfish infected by *A. hydrophila.*

## 5 Conclusion

To summarize, the purpose of this study was to assess the treat potential of baicalin to yellow catfish infected *A. hydrophila*. The *in vitro* results revealed that baicalin considerably restrained the growth of *A. hydrophila*, destroyed the bacterial cell wall, and reduced biofilm synthesis. The *in vivo* results demonstrated that administration of baicalin could improve the survival rate, effectively promote the antioxidant, anti-inflammatory capacity and improve the gut microbiota imbalance of yellow catfish against *A. hydrophila* challenge. As a result, baicalin can keep gut inflammatory homeostasis stable and boost the fuselage’s resistance to *A. hydrophila*. This study lays the groundwork for future research into the effects of baicalin in the field of antibacterial infection of aquaculture.

## Data Availability

The authors acknowledge that the data presented in this study must be deposited and made publicly available in an acceptable repository, prior to publication. Frontiers cannot accept a manuscript that does not adhere to our open data policies.
