# Supplementary material for: Baicalin enhances antioxidant, inflammatory defense, and microbial diversity of yellow catfish (Pelteobagrus fulvidraco) infected with Aeromonas hydrophila
Source: Front Microbiol. 2024 Sep 20;15:1465346. doi: 10.3389/fmicb.2024.1465346 (PMC11449889; doi:10.3389/fmicb.2024.1465346)
Supplement: Supplementary file 1 [file Table_1.DOCX]

Supplementary Table 1, Relative quantification of liver and intestinal villi

| Item | Control | Model | 10mg/kg | 15mg/kg | 25mg/kg | 35mg/kg |
| --- | --- | --- | --- | --- | --- | --- |
| Infiltration area of inflammatory cells (%) | 0.73± 0.08^d^ | 48.60±0.86^a^ | 44.62±3.77^b^ | 35.84±2.81^c^ | 36.10±1.81^c^ | 35.76±1.3^c^ |
| Intestinal villi (μm) | 276.23±20.50^c^ | 225.40±19.49^d^ | 305.50±7.59^bc^ | 308.00±15.56^bc^ | 327.00±19.03^ab^ | 340.75±25.02^a^ |

Note: All experiences were repeated three times and expressed as mean±SD, significant differences between groups with different superscripts a, b, c, and d (*P* <0.05).
